# Primary Febrile Neutropenia Prophylaxis for Patients Who Receive FEC-D Chemotherapy for Breast Cancer: A Systematic Review

**DOI:** 10.1200/JGO.2016.008540

**Published:** 2017-04-21

**Authors:** Ricardo Fernandes, Sasha Mazzarello, Carol Stober, Mohamed F.K. Ibrahim, Shaan Dudani, Kirstin Perdrizet, Habeeb Majeed, Lisa Vandermeer, Risa Shorr, Brian Hutton, Dean Fergusson, Bishal Gyawali, Mark Clemons

**Affiliations:** **Ricardo Fernandes**, **Mohamed F.K. Ibrahim**, **Shaan Dudani**, **Kirstin Perdrizet**, **Habeeb Majeed**, and **Risa Shorr**, The Ottawa Hospital; **Ricardo Fernandes**, **Shaan Dudani**, **Kirstin Perdrizet**, **Habeeb Majeed**, **Brian Hutton**, **Dean Fergusson**, and **Mark Clemons**, University of Ottawa; **Sasha Mazzarello**, **Carol Stober**, **Lisa Vandermeer**, **Brian Hutton**, **Dean Fergusson**, and **Mark Clemons**, Ottawa Hospital Research Institute, Ottawa, Ontario, Canada; and **Bishal Gyawali**, Nobel Hospital, Sinamangal, Kathmandu, Nepal.

## Abstract

**Purpose:**

Despite widespread use of fluorouracil, epirubicin, cyclophosphamide, docetaxel (FEC-D) chemotherapy in breast cancer, the optimal strategy for primary febrile neutropenia (FN) prophylaxis remains unknown. A systematic review was therefore performed.

**Methods:**

Embase, Ovid MEDLINE, PubMed, Cochrane Database of Systematic Reviews, Cochrane Register of Controlled Trials, and conference proceedings were searched from 1946 to April 2016 for trials that reported the effectiveness of primary FN prophylaxis with FEC-D chemotherapy. Outcome measures were incidence of FN; treatment-related hospitalizations; chemotherapy dose delays, reductions, and discontinuations; and adverse events from prophylaxis.

**Results:**

Of 2,205 identified citations, eight studies (n = 1,250) met our eligibility criteria. Three additional studies (n = 293) were identified from a prior systematic review. Three randomized controlled trials (n *=* 576), one phase IV single-arm trial (n = 69), one prospective observational study (n = 37), and six retrospective studies (n = 861) were identified. Agents investigated were pegfilgrastim (n = 108), filgrastim (n = 1,119), and ciprofloxacin (n = 89). The heterogeneity of studies meant that a narrative synthesis of results was performed. Median FN rates for patients who received FEC-D with and without primary prophylaxis were 10.1% (interquartile range [IQR], 3.9% to 22.6%) and 23.9% (IQR, 9.2% to 27.3%), respectively. In the absence of primary prophylaxis, FN was more common during docetaxel than during FEC. Data from six studies showed a median rate of dose reductions and delays of 6.1% (IQR, 3.1% to 14.3%) and 19.3% (IQR, 10.5% to 32.8%), respectively, that occurred as a consequence of FN. Toxicity from prophylaxis itself was rarely reported.

**Conclusion:**

Primary FN prophylaxis is effective in patients who receive FEC-D chemotherapy. The paucity of prospective data makes optimal recommendations about the choice and timing of prophylaxis challenging.

## INTRODUCTION

FEC-D (fluorouracil 500 mg/m^2^, epirubicin 100 mg/m^2^, cyclophosphamide 500 mg/m^2^, docetaxel 100 mg/m^2^) chemotherapy is an effective and commonly used regimen in the treatment of patients with early-stage breast cancer.^1-3^ At its usual doses and dosing intervals (three cycles of FEC once every 3 weeks followed by three cycles of docetaxel), febrile neutropenia (FN) is an important toxicity with this regimen. FN can be associated with considerable morbidity, mortality, and costs^4,5^ and often results in chemotherapy dose reductions, delays, and discontinuations.^6^ In the pivotal PACS 01 III trial where primary prophylactic granulocyte colony-stimulating factor (G-CSF) was administered in 22.2% of patients, FEC-D was associated with an 11.2% rate of FN.^3^ However, as its use expanded into routine clinical practice, reports of FN rates as high as 46.4% were reported.^7,8^ One systematic review identified that in routine clinical practice, FEC-D chemotherapy without G-CSF primary prophylaxis was associated with a median FN rate of 30.6% (95% CI, 26.8% to 34.6%). In contrast, for trials in the meta-analysis that used primary FN prophylaxis with G-CSF, the FN rate was 6.8% (95% CI, 4.4% to 10.0%).^8^

Most local,^9^ national,^10^ and international^11-13^ guideline groups have recommended that routine primary FN prophylaxis be used for regimens with an FN risk > 20%. Although consensus exists that FN prophylaxis should be recommended with FEC-D chemotherapy, no consensus has been reached on the optimal strategy (ie, either G-CSF or oral antibiotics) or timing (ie, from the start of chemotherapy or during the docetaxel component only) of such prophylaxis. These limitations are important given the considerable differences in cost and toxicity between agents.

To our knowledge, no high-quality evidence guides optimal FN prophylaxis with this commonly used chemotherapy regimen. Hence, we performed a systematic review to evaluate the incidence of FN with FEC-D (with and without primary prophylaxis), its timing, and optimal strategies for primary FN prophylaxis. We also identified gaps in the available literature that require further study.

## METHODS

### Study Objective and Eligibility Criteria

This systematic review was performed to identify and evaluate the incidence and timing of FN in the absence of primary FN prophylaxis and the effects of two strategies (G-CSF and antibiotics) for primary FN prophylaxis in patients who received six to eight cycles of FEC-D chemotherapy once every 3 weeks for early-stage breast cancer. The population, intervention, comparator, and outcome study design framework was used to structure the research question and its corresponding literature search. The population of interest was patients with breast cancer who received FEC-D chemotherapy in the adjuvant or neoadjuvant setting. Interventions of interest were primary FN prophylaxis G-CSF (ie, filgrastim, pegfilgrastim, biosimilars) and prophylactic antibiotics of any treatment duration. Primary FN prophylaxis was defined as prophylactic administration of hematopoietic cell growth factors (eg, G-CSF) or quinolone antibiotics to prevent the occurrence of infection. Comparators were best supportive care or prophylactic quinolone antibiotics. The primary outcome measure was the incidence of FN (defined as an absolute neutrophil count < 0.5 × 10^9^/L with oral temperature > 38.3°C or a temperature of > 38.0°C sustained over a 1-hour period). Secondary outcome measures were treatment-related hospitalizations; chemotherapeutic dose reductions, delays, and discontinuations; and frequency of adverse events from primary FN prophylaxis. Interventional or retrospective and prospective observational studies published in English were included. Animal studies, studies in the metastatic setting, and studies that involved patients who had received prior chemotherapy and secondary FN prophylaxis were excluded.

### Literature Search

An information specialist (R.S.) designed and executed an electronic literature search to identify relevant citations from Embase, Ovid MEDLINE, PubMed (including in-process and other nonindexed citations), the Cochrane Database of Systematic Reviews, the Cochrane Register of Controlled Trials, and conference proceedings from 1946 to April 26, 2016. Search terms and their medical subject heading equivalents are shown in the Data Supplement.

### Study Screening and Selection

**Fig 1:**
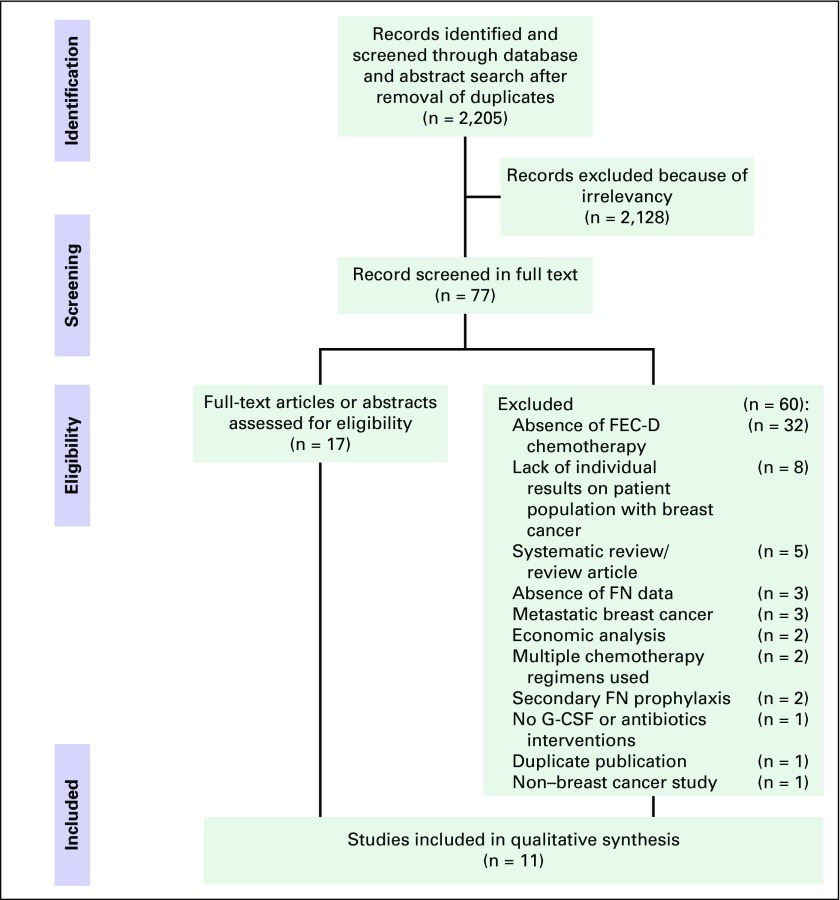
Preferred Reporting Items for Systematic Reviews and Meta-Analyses (PRISMA) flow diagram. FEC-D, fluorouracil, epirubicin, cyclophosphamide, docetaxel; FN, febrile neutropenia; G-CSF, granulocyte colony-stimulating factor.

Stage 1 screening consisted of a review of titles and abstracts identified from the literature search by two independent reviewers (R.F. and S.M.). Disagreements between the reviewers were discussed and resolved. A third reviewer was consulted if necessary to achieve consensus. Stage 2 screening consisted of a full-text review of all manuscripts and meeting abstracts from potentially relevant citations identified during stage 1 screening by two independent reviewers (R.F., S.M., C.S., M.F.K.I., S.D., K.P., L.V., or M.C.). We also reviewed the relevant studies included in a previous systematic review.^8^ The screening process is presented in a Preferred Reporting Items for Systematic Reviews and Meta-Analyses (PRISMA) flow diagram (Fig 1), and a PRISMA checklist was completed to document reporting elements of the review^17^ (Data Supplement).

### Data Collection and Risk of Bias Assessment

Data from the final set of included studies were extracted by two reviewers independently who used a predesigned form implemented in Microsoft Excel version 2010 (Microsoft Corporation, Redmond, WA); discrepancies were resolved by consensus discussion. The following information was collected from each study: publication characteristics (year, journal, and authors), patient characteristics (performance status, median age, and disease stage), intervention characteristics (chemotherapy regimen, neoadjuvant *v* adjuvant setting, type and frequency of G-CSF, and antibiotics used), and outcomes of interest (the incidence of febrile neutropenia; hospitalizations; chemotherapeutic dose reductions, delays, and discontinuations; and frequency of adverse events from primary FN prophylaxis). Authors were contacted to acquire other unpublished data. The Cochrane Collaboration’s tool for assessing risk of bias in randomized controlled trials was used^14^^,15^ (Data Supplement). Funding for the current study was from internal sources, and there was no pharmaceutical company funding.

### Data Analysis

If deemed appropriate, after exploration of study and patient characteristics to ensure sufficient clinical and methodological homogeneity across studies, we had planned to conduct meta-analyses using random-effects models to combine FN incidence data across studies. After inspection of the characteristics of the included studies, the research team determined a high degree of study heterogeneity in terms of study populations and design. We believed that these differences precluded the data from meta-analysis. To synthesize the information collected, a narrative summary was prepared to document incidence of FN and to summarize other related information, such as hospitalizations, the consequence of FN on chemotherapy, and adverse events.

## RESULTS

### Quantity of Evidence Identified

From 2,205 unique citations identified by the literature search, 77 potentially relevant studies were identified during the stage 1 screening of titles and abstracts. These 77 studies were subsequently reviewed in full text for stage 2 screening; eight of these studies met the eligibility criteria. In addition, three studies identified from a prior systematic review were included^8^ (Table 1). Reasons for study exclusion during stage 2 screening were absence of FEC-D chemotherapy use (n = 32), lack of individual results within the breast cancer population (n = 8), systematic review/review article (n = 5), absence of FN data (n = 3), metastatic breast cancer (n = 3), economic analysis (n = 2), multiple chemotherapy regimens used (n = 2), secondary FN prophylaxis (n = 2), no G-CSF or antibiotics interventions (n = 1), duplicate publication (n = 1), and non–breast cancer study (n = 1). Of the 11 included studies, eight were available as peer-reviewed manuscripts^16-21,24,25^ and three were available as meeting abstracts.^22,23,26^ The studies were published in 1998,^23^ 2003,^22^ 2008,^24^ 2009,^21^ 2010,^19,20,25^ 2011,^26^ 2012,^18^ 2013,^17^ and 2015.^16^

**Table 1:**
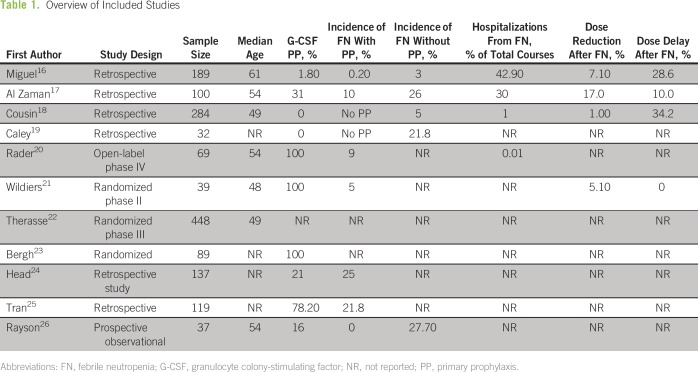
Overview of Included Studies

### Study Characteristics

Eligible studies included three randomized trials (n = 576), one phase IV single-arm trial (n = 69), one prospective observational study (n = 37), and six retrospective observational studies (n = 861). Sample sizes ranged from 32^19^ to 448^22^ patients. Pegfilgrastim was evaluated in two studies (n = 108),^20,21^ filgrastim in seven (n = 1,119),^16,17,22-26^ and ciprofloxacin in one (n = 89).^23^ None of the included studies reported use of interim neutrophil counts to guide G-CSF dosing.

Characteristics of the individual studies (study design; sample size; breast cancer stage; hormonal status; type of intervention; incidence of FN with and without primary prophylaxis; number of cycles delivered; number of patients and cycles with FN; incidence of hospitalizations; chemotherapeutic dose reductions, delays, and discontinuation; and frequency of adverse events) are listed in Table 1. As a result of considerable variability between studies in terms of study design and evaluated outcomes, meta-analysis was considered inappropriate, and a narrative summary as well as a descriptive overview of common results are presented. For example, few studies reported medical comorbidities, such as vascular disease, diabetes, and chronic obstructive pulmonary disease, known to affect FN rates.^20^

### FN Rates and Primary FN Prophylaxis

Overall, we found 1,543 patients treated with FEC-D (median age, 54 years; range, 24 to 78 years). FEC-D chemotherapy with and without primary prophylaxis was associated with median FN rates of 10.05% (range, 0.2% to 25%) and 23.9% (range, 5% to 27.7%), respectively.

With respect to each primary prophylaxis treatment, pegfilgrastim was used as a primary prophylaxis in two prospective studies.^20,21^ One study showed that among 32 patients treated with FEC-D who received primary FN prophylaxis with pegfilgrastim, FN developed in 5%.^21^ The other trial demonstrated that 9% of the 69 patients treated with FEC-D experienced FN despite receipt of primary prophylaxis with pegfilgrastim.^20^ Two studies provided detailed information about the use of primary prophylactic filgrastim.^16,17^ One trial demonstrated that of 100 patients treated with FEC-D, 26% and 10% had FN without and with filgrastim prophylaxis, respectively.^17^ In the other study, with a cohort of 189 patients treated with FEC-D, filgrastim was used in 1.8% and 9% during the FEC and docetaxel phases, respectively.^16^

### Timing of FN

Four studies reported the differences in FN rates between the FEC and docetaxel phases. In one study where 189 patients were treated with FEC-D, 7% and 21% experienced FN during the FEC and docetaxel phases, respectively.^16^ In another trial of 284 patients who had FEC-D without primary prophylaxis, 14 (4.9%) had FN. Overall, FN developed in 2.1% of patients during FEC cycles, whereas FN related to docetaxel occurred in 1.4% of patients.^18^ The third study comprised 32 patients treated with FEC-D without primary prophylaxis, where 9% and 21.8% experienced FN during the FEC and docetaxel phases, respectively.^19^ Finally, Rayson et al^26^ showed that among 37 patients without primary FN prophylaxis, the FEC and docetaxel cycles were associated with 35% and 62% FN rates, respectively. In summary, primary prophylaxis was only used in 9% to 24% of patients, and most episodes of FN occurred during docetaxel administration (interquartile range [IQR], 6.3% to 51.95%; median, 21.4%). In contrast, in the absence of primary prophylaxis, a median of 8% (IQR, 3.325% to 28.5%) of patients experienced FN during FEC cycles.^16,18,19,26^

### Risk Factors for FN

Traditional risk factors with a high/intermediate level of supporting evidence for FN are extensive prior chemotherapy; ≥ 85% relative intensity; age older than 65 years; poor performance status; low albumin/high lactate dehydrogenase levels; comorbidities such as pulmonary, cardiovascular, and liver disease; and diabetes mellitus.^9-12^ Primary prophylaxis was not used in all the included studies (median, 36.9%; range, 0% to 100%). FN risk factors were identified in two studies.^16,20^ In these two studies, the percentage of patients older than 65 years were 33.3%^16^ and 19%.^20^ Furthermore, 17% of patients were found to have medical comorbidities, such as vascular disease, diabetes, and chronic obstructive pulmonary disease.^20^ In one of the two studies that reported FN rates according to a 65-year age cutoff, the risk of developing FN during FEC was equal in patients older and younger than 65 years (risk ratio, 1.05; 95% CI, 0.51 to 2.2).^16^

### Consequences of FN

Data from six studies reported a median number of dose reductions and delays of 6.1% (IQR, 3.05% to 14.25%) and 19.3% (IQR, 10.5% to 32.8%) as a consequence of FN.^16-20,22^ Hospital admission as a result of FN occurred in 0.04% to 33.4% (median, 3%) of cases. The median duration of hospitalization reported was 6 days (range, 2 to 13 days).^16-18,20^ Only one study reported chemotherapy discontinuation rates as a result of FN that occurred in 6.7% of patients.^18^ Of note, across all the studies, no deaths occurred as a result of FN.

### Toxicity of Primary FN Prophylaxis

Few studies reported adverse effects of primary prophylaxis. Two studies with filgrastim reported primary prophylaxis toxicity, such as back pain (0.4%) and *Clostridium difficile* infection (7.6%).^17,20^

## DISCUSSION

FN is an important toxicity associated with FEC-D chemotherapy and can be associated with significant morbidity, mortality, and costs as well as a result of chemotherapy dose reductions, delays, and discontinuations. Because the proportion of FN cases in the absence of primary prophylaxis exceeds 20% with FEC-D chemotherapy, most guidelines^9-13^ recommend the use of primary FN prophylaxis. Primary prophylaxis is usually in the form of G-CSF or antibiotic use. However, despite the considerable differences in the cost and toxicity profiles of these agents as well as significant differences in the risk of FN depending on the chemotherapy received (docetaxel > FEC), we were unaware of high-quality data that compared either the choice of agent or its timing (during administration of FEC, docetaxel, or both).

The most effective strategy of providing FN prophylaxis is an important question not only to the physician and patients but also to the entire health care system in both the developed and the developing world because of its financial implications. Given the greater drug cost of pegfilgrastim over filgrastim, most health care funders will cover filgrastim, even though some data suggest that pegfilgrastim is superior and more cost-effective than filgrastim (Clinical Trials Information: NCT02173262).^27,28^ From a cost perspective alone, the cost differences are important from a global health care standpoint, with three cycles of FEC-D being associated with direct drug costs of $CAD1,740 for filgrastim for 10 days and $CAD2,422 for pegfilgrastim and $CAD35 for ciprofloxacin for 14 days.^9^ These costs do not include the charges for a health care professional to administer the G-CSF injections. Furthermore, from clinical experience, FN is much more commonly observed during the docetaxel component of treatment than during the FEC cycles. Finally, the toxicities of G-CSF differ from those of antibiotics as well as differ according to duration of use. Possible adverse effects of ciprofloxacin are nausea (> 2%) and, less commonly, diarrhea and vomiting (< 1%).^29^ Possible adverse effects of G-CSF (> 10%) are bone pain, headaches, irritation at the injection site, and diarrhea.^30^ The current systematic review attempts to address these questions from the synthesis of available evidence.

To our knowledge, this systematic review is the second to evaluate G-CSF and antibiotic use in patients with breast cancer who underwent FEC-D chemotherapy. Overall, median FN rates for patients who receive FEC-D with and without primary prophylaxis are 10.05% (IQR, 3.8% to 22.6%) and 23.9% (IQR, 9.2% to 27.2%), respectively. With respect to the timing of FN, four studies showed that most episodes occurred during docetaxel infusion cycles (median, 21.4; IQR, 6.3 to 51.9) compared with during FEC cycles (median, 8%; IQR, 3.3% to 28.5%).^16,18,19,26^

With respect to the choice of primary prophylaxis, pegfilgrastim (n = 108), filgrastim (n = 1,119), and ciprofloxacin (n = 89) were used. However, variable reporting of the use of different agents at different times (during FEC, docetaxel, or both) and as primary or secondary FN prophylaxis made it challenging to identify the optimal strategy. In fact, none of the included studies compared both strategies.

This systematic review had limitations. First, the included studies were mostly retrospective in design. Second, and of note, despite the widespread global use of FEC-D for more than a decade, a paucity of high-quality literature on the incidence, measurement, treatment, and prophylaxis of FN exists. The identified studies also lacked detailed and consistent outcome data, two of which were published in abstract form only, which leads to a risk of bias in these trials. Finally, although we aimed to compare two FN primary prophylaxis options (G-CSF and antibiotics), we were unable to find any such trial conducted previously.

Future studies are needed to determine the most effective treatment strategies to provide appropriate patient selection and individualized drug dosing and to prevent and reduce treatment-related toxicities. Only one clinical trial prospectively looked at optimal duration of filgrastim as FN primary prophylaxis, specifically in patients with early-stage breast cancer who underwent commonly used adjuvant chemotherapy regimens, including FEC-D.^28^ Unfortunately, no definitive results were available for antibiotic use. In addition, future trials could assess the timing of primary FN prophylaxis, for example, either from the start of FEC-D treatment or during the docetaxel component only. Robust economic analyses also are needed.

In conclusion, FN is a common toxicity of FEC-D chemotherapy. In light of the 20% FN threshold currently recommended for primary prophylaxis, the current results suggest that primary prophylaxis should be considered for the FEC-D regimen in routine clinical practice. Large population-based studies will help to clarify FN incidence in the real world, and randomized clinical trials are crucial to the establishment of treatment strategies and improvement of optimal G-CSF use.
